# RNF115 promotes lung adenocarcinoma through Wnt/β-catenin pathway activation by mediating APC ubiquitination

**DOI:** 10.1186/s40170-021-00243-y

**Published:** 2021-01-28

**Authors:** Xiao-Ting Wu, Yu-Han Wang, Xiao-Yue Cai, Yun Dong, Qing Cui, Ya-Ning Zhou, Xi-Wen Yang, Wen-Feng Lu, Ming Zhang

**Affiliations:** 1 Department of Integrated Traditional Chinese and Western Medicine, Shanghai Chest Hospital, Shanghai Jiao Tong University, No. 241 West Huaihai Road, Xuhui District, Shanghai, 200030 China; 2 Department of Integrated Traditional Chinese and Western Medicine, Zhongshan Hospital, Fudan University, Shanghai, China; 3 LongHua Hospital Shanghai University of Traditional Chinese Medicine/Oncology Division 2, Shanghai, China

**Keywords:** Cell proliferation, Glycolysis, Apoptosis, Wnt pathway, Ubiquitination

## Abstract

**Background:**

Patients with lung adenocarcinoma (LUAD) have high mortality rate and poor prognosis. The LUAD cells display increased aerobic glycolysis, which generates energy required for their survival and proliferation. Deregulation of Wnt/β-catenin signaling pathway induces the metabolism switching and oncogenesis in tumor cells. RING finger protein 115 (RNF115) is an E3 ligase for ubiquitin-mediated degradation. Although the oncogenic functions of RNF115 have been revealed in breast tumor cells, the effect of RNF115 on lung cancer is still not clear.

**Methods:**

RNF115 expression and its correlation with the features of LUAD patients were analyzed by using public database and our own cohort. The functions of RNF115 in proliferation and energy metabolism in LUAD cells were explored by downregulating or upregulating RNF115 expression.

**Results:**

We demonstrated that RNF115 was overexpressed in LUAD tissues and its expression was positively correlated with the poor overall survival of LUAD patients. Moreover, RNF115 overexpression inhibited LUAD cell apoptosis and promoted cellular proliferation and metabolism in LUAD cells. On the contrary, RNF115 knockdown displayed reverse effects. Furthermore, the underlying mechanism of the biological function of RNF115 in LUAD was through regulating Wnt/β-catenin pathway via ubiquitination of adenomatous polyposis coli (APC).

**Conclusion:**

The current study reveals a close association between RNF115 expression and prognostic conditions in LUAD patients and the oncogenic roles of RNF115 in LUAD at the first time. These findings may help establish the foundation for the development of therapeutics strategies and clinical management for lung cancer in future.

**Supplementary Information:**

The online version contains supplementary material available at 10.1186/s40170-021-00243-y.

## Background

Non-small cell lung cancer (NSCLC) is heterogeneous and the most prevailing lung cancer subtype, accounting for 80–85% of all the lung cancer cases [1]. As one type of NSCLCs, lung adenocarcinoma (LUAD) is currently the most common form of lung cancer with the largest number of mutations, relative high mortality rate, and poor prognosis, and the initiation and progression of LUAD is driven by recurrent somatic alterations [2, 3]. The LUAD tumor cells display metabolic remodeling, favoring the endogenous fatty acids metabolism and aerobic glycolysis, which generates enormous amounts of energy required for their survival and proliferation, instead of the standard mitochondrial respiration performed by normal healthy cells [4–6]. Common treatments for LUAD at different stages mainly rely on surgical resection, radiotherapy, chemotherapy, and immunotherapy [1, 7], whereas, tumor recurrence and further metastasis, as a result of the tumor resistance, frequently happen in LUAD patients after diagnosis and relevant treatment, leading to the low efficacy of therapy and poor clinical management [8, 9].

Wnt signaling pathway is one of the most critical cascades regulating endogenous stem cells and participating in tissue homeostasis and development [10]. Aside from the normal regulation on cell differentiation and proliferation, Wnt signaling pathway is also known for its capability in induction of malignant cell development and influencing the metabolic remodeling in tumor, once aberrantly regulated or being out of control by its regulators [11, 12]. Although the detailed network of which Wnt pathway triggers metabolism switching into dominant glycolysis is still under study, several common downstream signaling pathways were identified to be modulated by Wnt signaling pathway and associate with direct metabolic remodeling in tumorous cells, including NSCLC cells [13]. These include but are not limited to TCF/LEF, c-myc, and Akt-mTOR pathways [14]. On the other hand, Wnt signaling pathway is also able to indirectly influence the cellular metabolic processes through regulating relevant oncogenes and rate-limiting enzymes [14].

The canonical Wnt pathway signals through the core molecule β-catenin, with multiple other major signal transducers identified as well, including adenomatous polyposis coli (APC), Axis inhibition protein (AXIN), and glycogen synthase kinase 3β (GSK-3β) [15]. At normal situation, β-catenin is sustained at a relatively low level by ubiquitin proteasome system with the absence of Wnt signal [16]. For this system, AXIN, APC, and GSK-3β form a destruction complex, recruiting the accumulated β-catenin in cytosol and facilitating its phosphorylation for subsequent degradation [17]. The ubiquitination and proteasomal degradation of β-catenin contribute to restricting its cytoplasmic accumulation, whereas aberrant control of β-catenin leads to its massive translocation from cytoplasm into the nucleus of the cells, in which it binds and collaborates with various oncogene regulators to further induce oncogenesis in the cells [15, 17, 18].

RING finger protein (RNF) family, possessing intrinsic E3 ligase capability, is actively involved in the tumorigenesis of human cancers through ubiquitin-mediated degradation of oncoproteins [19, 20]. Previous study has reported that a novel RNF115, whose E3 ligase activity is stabilized by ubiquitin-specific protease 9X, is overexpressed in breast carcinomas [21, 22]. The expression of RNF115 significantly affects the cell growth and progression in breast cancer, and it is substantially correlated with estrogen receptor positive status [21]. As a matter of fact, RNF115 is encoded by breast cancer-associated gene 2 (BCA2) [23, 24], which gene is later identified as the transcriptional target and direct downstream gene of estrogen receptor α [25]. Moreover, RNF115 has been demonstrated to accelerate the breast cancer cell proliferation possessing estrogen receptor α partly by suppressing p21 expression via ubiquitin-mediated degradation [26]. However, information about the function of RNF115 in lung cancer, especially in LUAD, is still limited.

In the current study, we aimed to explore the correlation between RNF115 and LUAD prognosis, the functions of RNF115 on tumor cell proliferation and metabolic remodeling in LUDA, and their potential underlying mechanisms. We reveal that RNF115 is highly expressed in LUAD cells and its expression is negatively correlated with the survival probability in LUAD patients. Furthermore, we also demonstrate that RNF115 can suppress LUAD cell apoptosis and stimulate its proliferation, cellular respiration, and glycolytic process, possibly through motivating Wnt/β-catenin pathway by catalyzing APC ubiquitination.

## Methods

### Bioinformatics analysis

The LUAD dataset was downloaded from the website of The Cancer Genome Atlas (TCGA) database (https://tcga-data.nci.nih.gov/tcga/). The analyses of gene expression were conducted by Gene Set Enrichment Analysis (GSEA) based on the TCGA LUAD dataset. The Kaplan-Meier Plotter website (www.kmplot.com) was applied for examining the prognostic values of RNF115 mRNA expression [27].

### Study subjects

The 1975 Declaration of Helsinki ethical guidelines were followed for designing our study protocol, and the final protocol was permitted by the Institutional Ethical Review Committee of Shanghai Chest Hospital, Shanghai Jiao Tong University (Shanghai, China). All lung adenocarcinoma (LUAD) patients with surgery between January 2007 and December 2008 at Shanghai Chest Hospital, Shanghai Jiao Tong University (Shanghai, China) were enrolled for this study, and they were provided with the informed consents. LUAD tumor tissues and their normal adjacents were collected from 25 patients (cohort 1). Following surgical resection, these tissues were immediately frozen by liquid nitrogen, and then they were kept at − 80 °C. Specimens from 80 LUAD patients (cohort 2) were collected for IHC analysis. The clinical information of these 80 patients was obtained by reviewing their medical records. The follow-up study period lasted 5 years. The patients who underwent radiotherapy or immunotherapy along with surgery were excluded.

### Quantitative real-time PCR (qRT-PCR)

Trizol reagent (Invitrogen, USA) was used for the extraction of total RNA based on the instructions from the manufacturer. The transcriptional levels of RNF115 were examined by qRT-PCR by Applied Biosystems 7300 instrument (ABI, USA) using SYBR®Green reagent (Thermo Fisher Scientific, China), with GAPDH as the housekeeping gene for normalization. The primers used for quantify target gene expressions included: RNF115, 5′-TTGAAAGCCAAGACACAAG-3′ and 5′-ACTGCCCAAGTTTATGAAG-3′; APC, 5′-AAATGTCCCTCCGTTCTTATG-3′ and 5′-TCTGAAGTTGAGCGTAATACC-3′; and GAPDH, 5′-AATCCCATCACCATCTTC-3′ and 5′-AGGCTGTTGTCATACTTC-3′. The qRT-PCR program was carried out with initial 95 °C for 10 min and 40 cycles of 15 s at 95 °C for and 45 s at 60 °C. The specific amplicon was verified by the analysis based on dissociation curve. The comparison and quantification of gene expressions were performed by comparative Ct method. The formula of 2^−ΔΔCT^ was used for determination of the fold-change of target genes, after GAPDH normalization, and presented as the mean values. All experiments were performed by three replicates.

### Immunohistochemistry (IHC) analysis

The LUAD specimens were deparaffinized by xylene, rehydrated in ethanol, and harvested through antigen association in citrate buffer (0.01 M, pH 6.0) by microwaving for 15 min. To deactivate endogenous peroxidases, the retrieved sections were further reacted with 0.3% hydrogen peroxide by 30 min incubation, followed by incubation in 10% goat serum for another 30 min. Following the overnight association with antibody against RNF115 (Bioss Inc., USA) or β-catenin (Abcam, USA) at 4 °C, the complexes were further incubated for 1 h with a secondary antibody with horseradish peroxidase (HRP) conjugation at room temperature. The immunoreactivity was visualized by 3,3-diaminobenzidine (DAB) solution staining and hematoxylin counterstaining. Then these specimens were grouped into high (> 20% positive cells) and low (< 20% positive cells) expressions based on the RNF115 immunoreactivity grading.

### Cell culture

Human embryonic kidney cell line (293T), human bronchial epithelial cell line (16HBE), and human LUAD cell lines, including A549, CALU1, H358, H1299, and H1975, were obtained from the Shanghai Cell Bank (Chinese Academy of Sciences, China). All the cells were cultured in Dulbecco’s modified Eagle medium (DMEM) supplemented with 10% fetal bovine serum (FBS) and 100 μg/mL streptomycin-penicillin solution at the standard condition (5% CO_2_, 37 °C, and 85–95% humidity).

### Manipulation of RNF115 expression by lentivirus and cell transfection

The sequences of short hairpin RNA (shRNA) oligos targeting RNF115, for amplification and cloning into pLKO.1 vector (Addgene, USA) with AgeI/EcoRI digestion, were as follows: 1#, 5′-CCAGATGTGAATCAGGCTT-3′; 2#, 5′-CCAAGATAATAGAGCCAAT-3′; and 3#, 5′-GGGCTTGATGCCATTGTAA-3′. Meanwhile, the complete human RNF115 gene was inserted in pLVX-puro vector (Clontech, USA).

The lentiviruses for pLVX-RNF115 (oeRNF115), pLVX-puro (Vector), RNF115 shRNA (shRNF115), or control shRNA (shNC) was expressed in 293T cells with the presence of pMD2.G and psPAX2 as the packaging plasmids. H358 and H1975 cells were transfected with shRNF115 (1# and 2#) or shNC for control, and H1299 cells were transfected with oeRNF115 or vector for control, followed by treating with 10 μmol/L XAV939 (Sigma, USA) for further analysis.

### Overexpression of β-catenin

Full length human β-catenin gene was inserted into pCDNA3.1 vector (Invitrogen, USA). H358 and H1975 cells were transfected with pCDNA3.1-β-catenin (oeβ-catenin) or pCDNA3.1 vector for control.

### Western blotting analysis

Cell lysis was conducted by using radio-immunoprecipitation buffer (Beyotime Biotechnology, China) with proteinase inhibitor. The cytosolic or nuclear cell fraction was extracted by using NE-PER™ Nuclear and Cytoplasmic Extraction Reagents (Thermo Fisher Scientific, USA). Sodium dodecyl sulfate-polyacrylamide gel electrophoresis (SDS-PAGE) was performed for separating proteins in the cell fractions, followed by nitrocellulose membrane electroblotting (Millipore, USA). The membranes were blocked in 5% skim milk and overnight incubated with primary antibodies (Table S1) at 4 °C. After the unbound antibody was washed away, the membranes were further incubated with the HRP-conjugated secondary antibody mentioned above for another 1 h at room temperature. The signal detection was conducted by enhanced chemiluminescence system (ECL) (Millipore, USA).

### Cell Counting Kit-8 assay and Click-iT EdU assay

Cell Counting Kit-8 (CCK-8, Dojindo Laboratories, Japan) and Click-iT EdU Cell Proliferation Assay were utilized for detecting cellular proliferation in LUAD cells. For CCK-8 assay, approximately 2 × 10^3^ cells seeded in individual well of the 96-well plates were underwent viral infection for 24, 48, and 72 h. Then, the cells in each individual well were reacted with 10 μl of CCK-8 followed by 1 h incubation at 37 °C. A multiskan MS plate reader (Labsystems, Finland) was utilized for the assessment of viable cell numbers based on their absorbance at the wavelength of 450 nm.

Click-iT EdU flow cytometry Kit (US Everbright Inc, China) was used for EdU incorporation assay. Approximately 2 × 10^5^ cells seeded in individual well of the 6-well plates were underwent viral infection for 48 h. Then, the cells in each individual well were labeled with 50 μM EdU for 2 h prior to harvesting. After collected, the cells were fixed with 4% paraformaldehyde, permeabilized with 0.5% Triton X-100, stained with freshly prepared staining solution, and analyzed with flow cytometry (BD Biosciences, USA) according to the manufacturer’s protocol.

### Evaluation of cellular apoptosis

The harvested cells were washed with pre-chilled phosphate buffered saline followed by annexin V staining using fluorescein isothiocyanate (FITC) apoptosis detection kit (KeyGEN Biotech, China) based on the instruction from the manufacture. A flow cytometer (BD Biosciences, USA) was used for analyzing the rate of apoptosis in the cells.

### Mice model for xenograft

The protocol for animal trials was approved by the Animal Care Committee of Shanghai Chest Hospital, Shanghai Jiao Tong University (Shanghai, China). Ten 4-week-old male nude mice were obtained from Shanghai Laboratory Animal Center, China, and randomly separated into experimental and control groups (*n* = 5 per group), and subcutaneously injected (5 × 10^6^ cells per mouse) with H358 cells which stably express shRNF115#1 (#1) or control shRNA (NC), or H1299 cells which stably express RNF115 (oRNF115) or Vector. The volume of tumor was monitored every third day and calculated as follows: 0.5 × (the largest diameter) × (the smallest diameter)^2^. Then, at the 33rd day, the recovery of xenografts was performed following mice euthanization, and the tumor wet weight was measured. The xenografts were processed for TdT-mediated DUTP nick end labeling (TUNEL) as well as western blotting analysis.

### Measurement of energy metabolism in cells

The oxygen consumption rate (OCR) and extracellular acidification rate (ECAR) were measured using XF96 extracellular flux analyzer (Seahorse Bioscience, USA) based on the instructions from the manufacturer. Approximately 4 × 10^4^ cells were plated in individual well of the microplates 1 day before the measurement. For ECAR, the glyco-stress test kit (Seahorse Bioscience, USA), 10 mM of glucose, 50 mM of 2-[N-(7-nitrobenz-2-oxa-1,3-diazol-4-yl) amino]-2-deoxyglucose, and 2 μM of oligomycin were used. For OCR, the mito-stress kit (Seahorse Bioscience, USA), 1.5 μM of fluoro-carbonyl cyanide phenylhydrazone, 2 μM oligomycin, and pre-mixed 1 μM of antimycin A with 100 nM of rotenone were used.

### Co-immunoprecipitation (co-IP)

Cell lysates were incubated with antibody against RNF115 (Novus Biologicals, Inc., USA), anti-APC antibody (Beyotime Biotechnology, China), or reference control IgG (Santa Cruz Biotech., USA) at 4 °C for 1 h, followed by 3 h mixing with 150 μg protein A/G-agarose in 4 °C. The harvested precipitates were washed in radio-immunoprecipitation buffer (Beyotime Biotechnology) for three times for following western blotting analysis.

### Statistical analysis

The data statistical analyses were conducted by Graphpad Prism version 6.0, (San Diego, USA). Both the analysis of variance and Student’s *t* test were applied for comparing the data. The significance was determined at the significant level of 0.05.

## Results

### Higher expression of RNF115 in LUAD was correlated with its prognosis

**Fig. 1:**
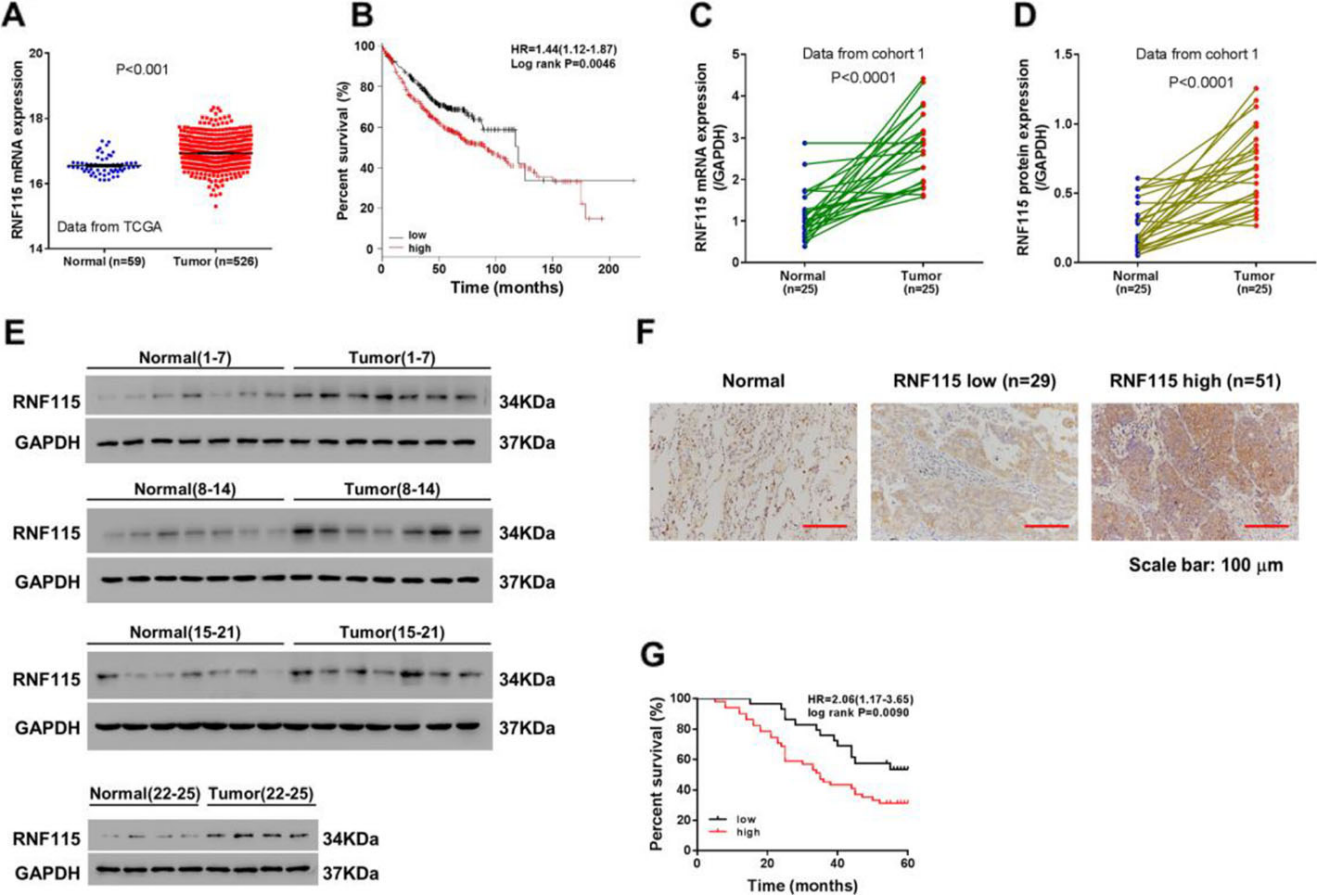
Association between RNF115 expression and LUAD prognosis. **a** Expressions of RNF115 in normal or LUAD tumorous tissues were analyzed based on TCGA database. **b** Comparison of the survival probability between LUAD patients with high and low expressions of RNF115 over 200 months by Kaplan-Meier plotting. **c**–**e** Analysis of RNF115 gene expression in 25 paired tumorous and adjacent non-tumorous tissues of LUAD patients by qRT-PCR (**c**) and western blotting (**d**, **e**). **f** Immunohistochemistry staining and analysis of the levels of RNF115 in tumorous and neighboring normal tissues from LUAD patients. Scale bar, 100 μm. **g** Kaplan-Meier plot for survival rate of LUAD patients with differential RNF115 expression (high or low) over 60 months

The expression levels of RNF115 in the tissues collected from LUAD patients and their correlation with prognostic features of LUAD patients were analyzed dependent on the data collected from TCGA database (Fig. 1a, b). In details, the average expression level of RNF115 in the 59 normal tissues was significantly (*P* < 0.001) lower than that in the 526 tumorous tissues in LUAD patients (Fig. 1a). At the same time, the high expression of RNF115 was found to be significantly correlated (HR = 1.44, *P* = 0.0046) with the low survival probability of LUAD patients after relevant therapy (Fig. 1b). Furthermore, through qRT-PCR (Fig. 1c) and western blotting analysis (Fig. 1d, e) based on paired tumorous and adjacent non-tumorous tissue sections collected from 25 LUAD patients (cohort 1), we also detected that RNF115 was significantly (*P* < 0.001) upregulated in the tumorous tissues in comparison with that in the neighboring non-tumorous tissues. In fact, the protein expression levels of RNF115 were also higher in LUAD cell lines, including A549, CALU1, H358, H1299, and H1975, in relation to that in 16HBE cells (Figure S1A).

On the other hand, relying on IHC analysis, we have detected 29 cases of low RNF115 expression and 51 cases of high expression in total from the tumor specimens collected from 80 LUAD patients (cohort 2) (Fig. 1f). Additionally by analyzing the correlation between high/low RNF115 expression and the features of these 80 patients, we have revealed a significant (*P* = 0.0104) correlation between the RNF115 expression and the tumor size in the patients, whereas insignificant (*P* > 0.05) correlations were found between RNF115 expression and other individual feature of the patients, such as gender, age, differentiation, and status of lymph node metastasis (Table 1). The survival rate of these 80 patients was significantly (HR = 2.06, *P* = 0.0090) correlated with the level of RNF115 expression as well (Fig. 1g).

**Table 1:** Correlation of RNF115 expression with features of LUAD patients

Variables		All cases	RNF115 protein		***P*** value
			Low (***n*** = 29)	High (***n*** = 51)	
Gender	Male	44	18	26	0.3609
	Female	36	11	25	
Age (years)	≥ 60	39	11	28	0.1683
	< 60	41	18	23	
Differentiation	Well	13	5	8	0.7089
	Moderate	46	15	31	
	Poor	21	9	12	
Tumor size (cm)	≥ 5	48	12	36	0.0104*
	< 5	32	17	15	
Lymph node metastasis	Positive	37	10	27	0.1114
	Negative	43	19	24	

*P < 0.05

### RNF115 promoted cell proliferation and inhibited cell apoptosis in LUAD

**Fig. 2:**
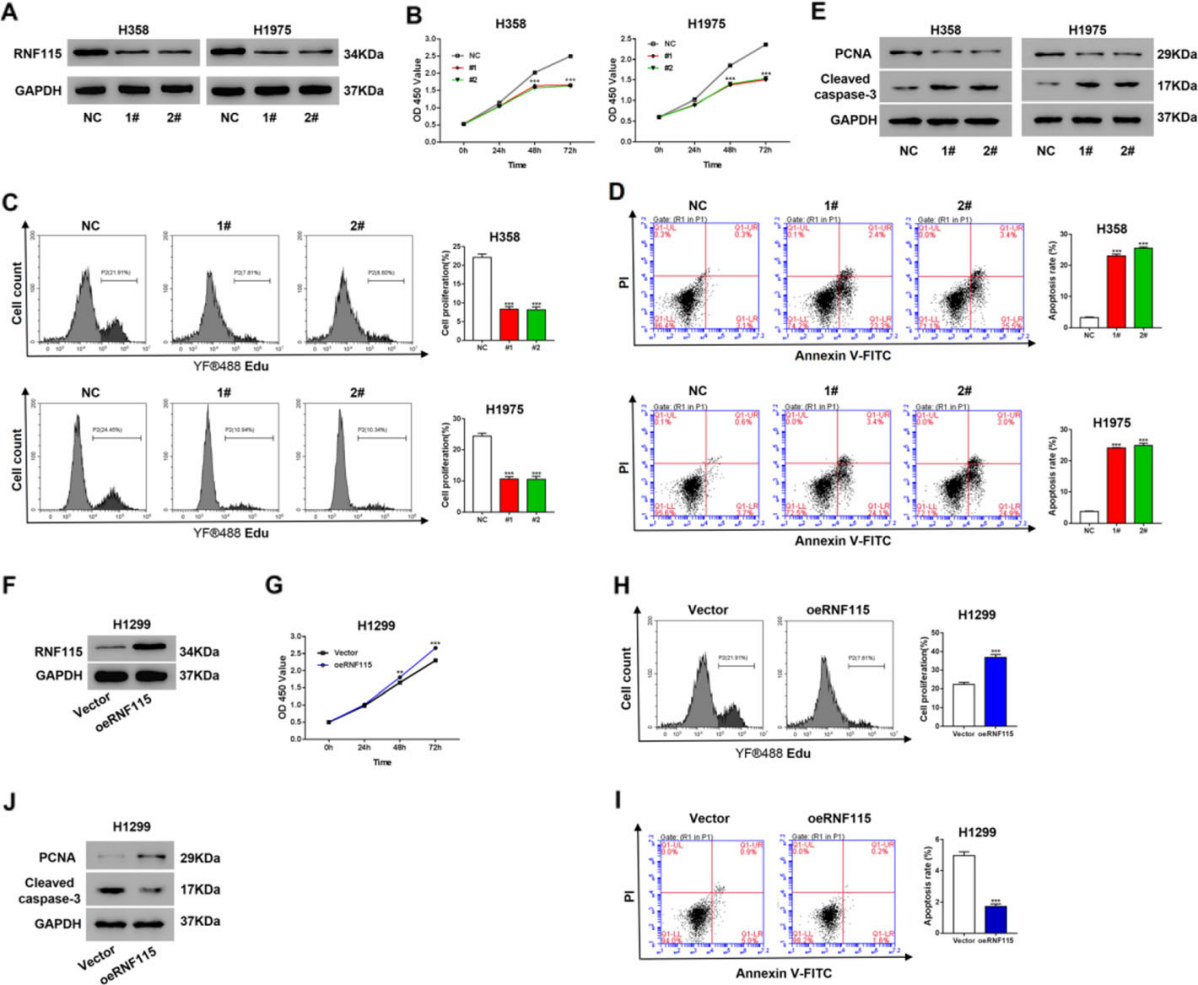
Effectiveness of RNF115 expression on proliferation and apoptosis in LUAD cells. **a**–**e** Comparison of RNF115 expression, cellular proliferation, and apoptosis between H358 or H1975 cells transfected with shRNF115 (1# and 2#) and control shRNA (NC). **a** Expression of RNF115 protein by western blotting with the loading control as GAPDH. **b**, **c** Measurement of cell proliferation by CCK-8 assay (**b**) and Click-iT EdU Cell Proliferation Assay (**c**). **d** The ratio of apoptotic cells. ***P < 0.001 vs NC. **e** Expression of PCNA and Cleaved caspase-3 by western blotting. **f**–**i** Comparison of RNF115 expression, cellular proliferation, and apoptosis between H1299 cells transfected with oeRNF115 and Vector control. **f** Expression of RNF115 protein by western blotting with the loading control as GAPDH. **g**, **h** Measurement of cell proliferation by CCK-8 assay (**g**) and Click-iT EdU Cell Proliferation Assay (**h**). **i** The ratio of apoptotic cells. **P < 0.01 and ***P < 0.001 vs Vector

For examining the effectiveness of RNF115 on tumor cell proliferation and apoptosis, we have established two types of LUAD cells: (1) RNF115 knockdown in the cell lines (H358 and H1957) with relatively higher expression of RNF115 (Figs. 2a and S1B) and (2) RNF115 overexpression in the cell line (H1299) with relatively lower expression of RNF115 (Figs. 2f and S1C). RNF115 knockdown (both shRNF115 1# and 2#) significantly (*P* < 0.001) reduced the cell proliferation of H358 and H1975 cells as indicated by CCK-8 (Fig. 2b) and Click-iT EdU Cell Proliferation Assay (Fig. 2c), while RNAi-resistant mutant of RNF115 could rescue proliferation inhibition caused by RNF115 knockdown (Figure S2B). Additionally, RNF115 knockdown (both shRNF115 1# and 2#) significantly (*P* < 0.001) increased the cell apoptotic rates of H358 and H1975 (Fig. 2d). RNF115 knockdown (both shRNF115 1# and 2#) decreased the expression of the proliferation marker, PCNA, and increased the expression the apoptosis marker, Cleaved caspase-3(Fig. 2e). On the contrary, RNF115 overexpression (oeRNF115) in H1299 cells significantly elevated cell proliferation (Fig. 2g, h) and the expression of PCNA (Fig. 2j), inhibited the cell apoptotic rate (Fig. 2i) and the expression of Cleaved caspase-3 (Fig. 2j).

### RNF115 stimulated tumor growth and suppressed tumor cell apoptosis in mice with LUAD induction

**Fig. 3:**
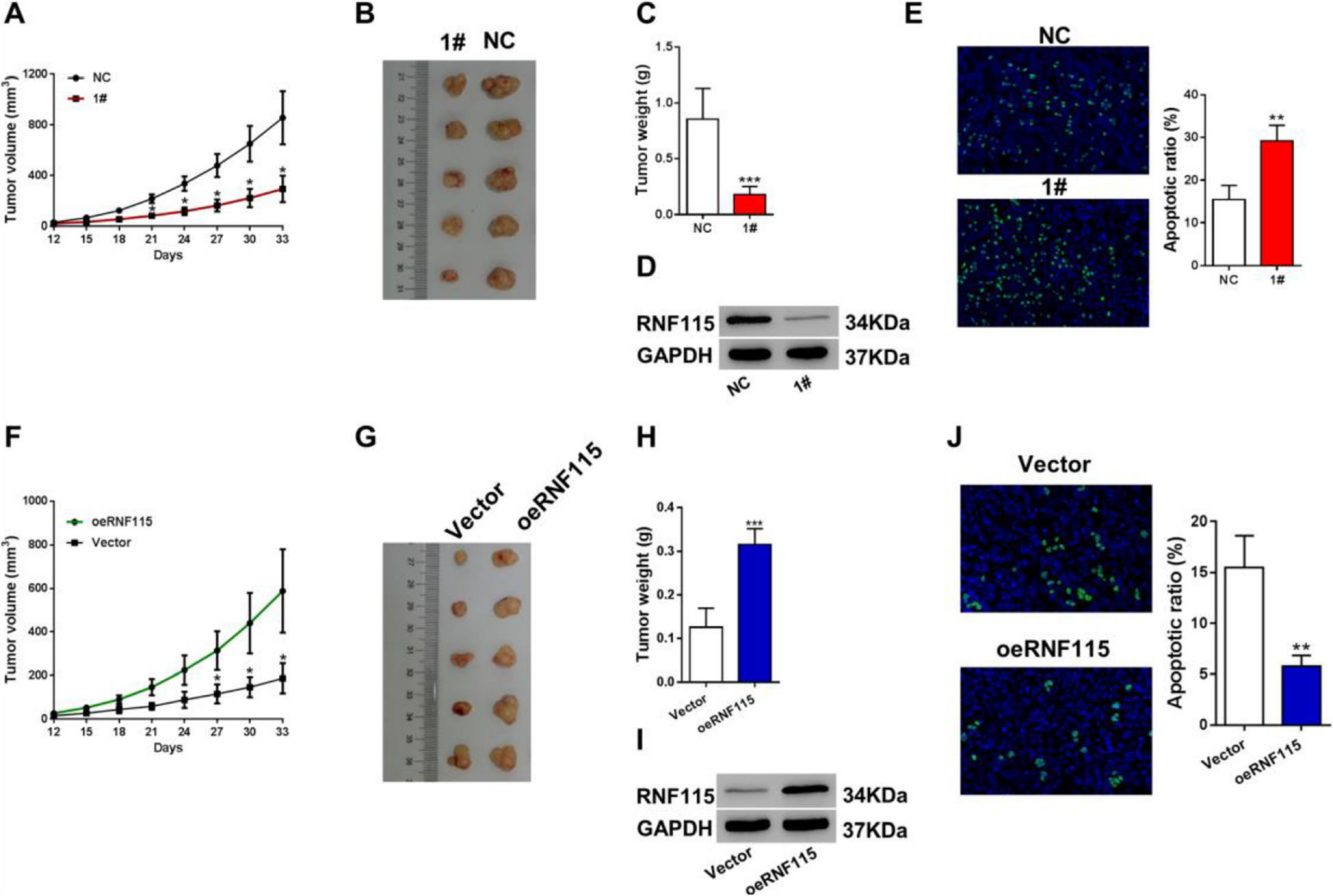
Function of RNF115 expression on xenograft growth in mice. **a**–**e** Comparison of RNF115 expression and xenograft status between mice (*n* = 5 per group) subcutaneously injected with H358 cells stably expressing shRNF115 (1#) and control shRNA (NC). **a** Tumor volume growth in mice after injection. **b** Image and comparison of the xenografts at day 33. **c** Tumor weights at day 33. **d** Expression of RNF115 protein in the xenografts at day 33 by western blotting with the loading control as GAPDH. **e** Visualization and analysis of the apoptotic cells in the xenografts at day 33 by TUNEL. Images at × 200 magnification. *P < 0.05, **P < 0.01, and ***P < 0.001 vs NC. **f**–**j** Comparison of RNF115 expression and xenograft status between mice (*n* = 5 per group) subcutaneously injected with H1299 cells stably expressing RNF115 (oRNF115) and control (Vector). **f** Tumor volume growth in mice after injection. **g** Image and comparison of the xenografts at day 33. **h** Tumor weights at day 33. **i** Expression of RNF115 protein in the xenografts at day 33 by western blotting. GAPDH as loading control. **j** Visualization and analysis of the apoptotic cells in the xenografts at day 33 by TUNEL. Images at × 200 magnification. *P < 0.05, **P < 0.01, and ***P < 0.001 vs Vector

In order to determine the functional roles of RNF115 in tumor cells in vivo, we have established two types of mice models: (1) H358 cells with RNF115 knockdown (shRNF115) subcutaneously injected mice and (2) H1299 cells with RNF115 overexpression (oeRNF115) subcutaneously injected mice. The tumor volume was found to be significantly (*P* < 0.05) smaller in shRNF115 cells (1#)-injected mice during days 21–33 compared with the control group (Fig. 3a, b), whereas it was significantly (*P* < 0.05) larger in oeRNF115 cells-injected mice during days 27–33 in relation with the control group (Fig. 3f, g). Similarly, compared to the control at day 33, RNF115 knockdown (shRNF115 1#) significantly (*P* < 0.001) decreased the tumor weight in mice (Fig. 3c), while RNF115 overexpression (oeRNF115) significantly (*P* < 0.001) increased the tumor weight in mice (Fig. 3h). The knockdown (Fig. 3d) and overexpression of RNF115 (Fig. 3i) was confirmed in xenografts by western blotting. In addition, through TUNEL, we observed that the rate of tumor cell apoptosis in mice was significantly (*P* < 0.01) elevated by RNF115 knockdown (shRNF115 1#) (Fig. 3e) but significantly (*P* < 0.01) suppressed by RNF115 overexpression (oeRNF115) (Fig. 3j).

### RNF115 knockdown reduced metabolic processes and inhibited β-catenin pathway in LUAD cells

**Fig. 4:**
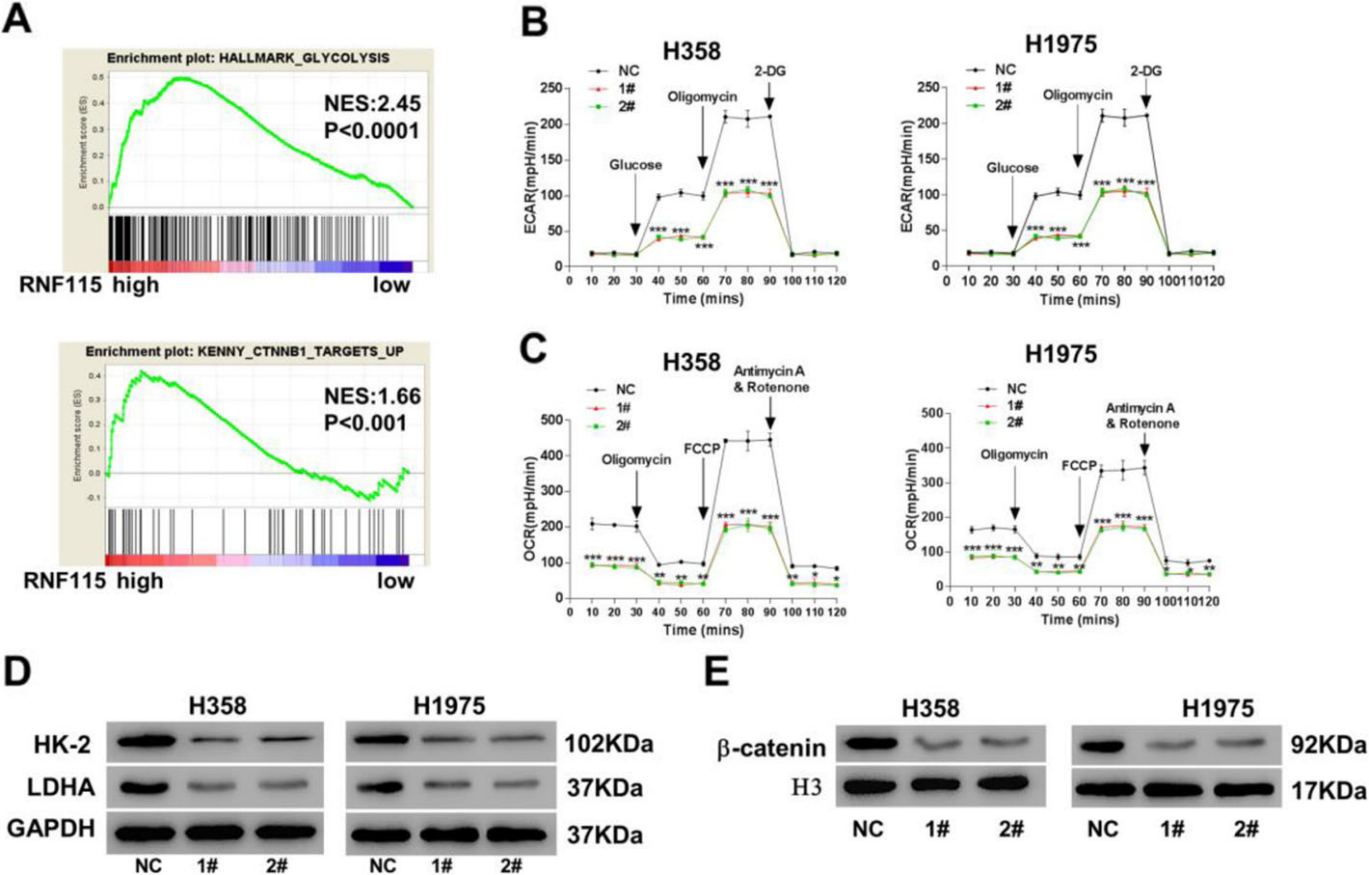
Effects of RNF115 knockdown in cellular metabolism of LUAD. **a** Correlation between the expression of RNF115 and glycolytic activity (upper panel) or β-catenin pathway (lower panel) by GSEA based on TCGA database. **b**–**e** Comparison of energy metabolism and related protein expressions between H358 or H1975 cells transfected with shRNF115 (1# and 2#) and control shRNA (NC). **b** Extracellular acidification rates for glycolysis. **c** Oxygen consumption rates for mitochondrial respiration. **d** Expressions of HK-2 and LDHA proteins in cytosol by western blotting with the loading control as GAPDH. **e** Expression of β-catenin in nucleus by western blotting with the loading control as H3. *P < 0.05, **P < 0.01, and ***P < 0.001 vs NC

For exploring the function of RNF115 in tumor cell metabolism, the correlation between high/low expression of RNF115 and glycolytic process or β-catenin pathway was analyzed by GSEA. We found that RNF115 expression was considerably and positively correlated with both glycolytic process (*P* < 0.001) and β-catenin pathway (*P* < 0.01) in LUAD (Fig. 4a). Moreover, RNF115 knockdown (both shRNF115 1# and 2#) significantly (*P* < 0.001) suppressed the glycolytic activities, including glycolysis and glycolytic capacity, in both H358 and H1975 cells (Fig. 4b). Following the similar pattern, RNF115 knockdown (both shRNF115 1# and 2#) also significantly suppressed the mitochondrial respiration, in both H358 and H1975 cells (Fig. 4c).

Furthermore, based on western blotting analysis, we observed that the glycolysis-related proteins hexokinase-2 (HK-2) and lactate dehydrogenase-A (LDHA) were downregulated in H358 and H1975 cells with RNF115 knockdown (both shRNF115 1# and 2#), when they were compared to those in the control cells (Fig. 4d). In relation with the control, the protein levels of β-catenin were also downregulated in the nucleus of H358 and H1975 cells with RNF115 knockdown (both shRNF115 1# and 2#) (Fig. 4e).

### RNF115 regulated LUAD cell activities by modulating Wnt/β-catenin pathway through APC ubiquitination

**Fig. 5:**
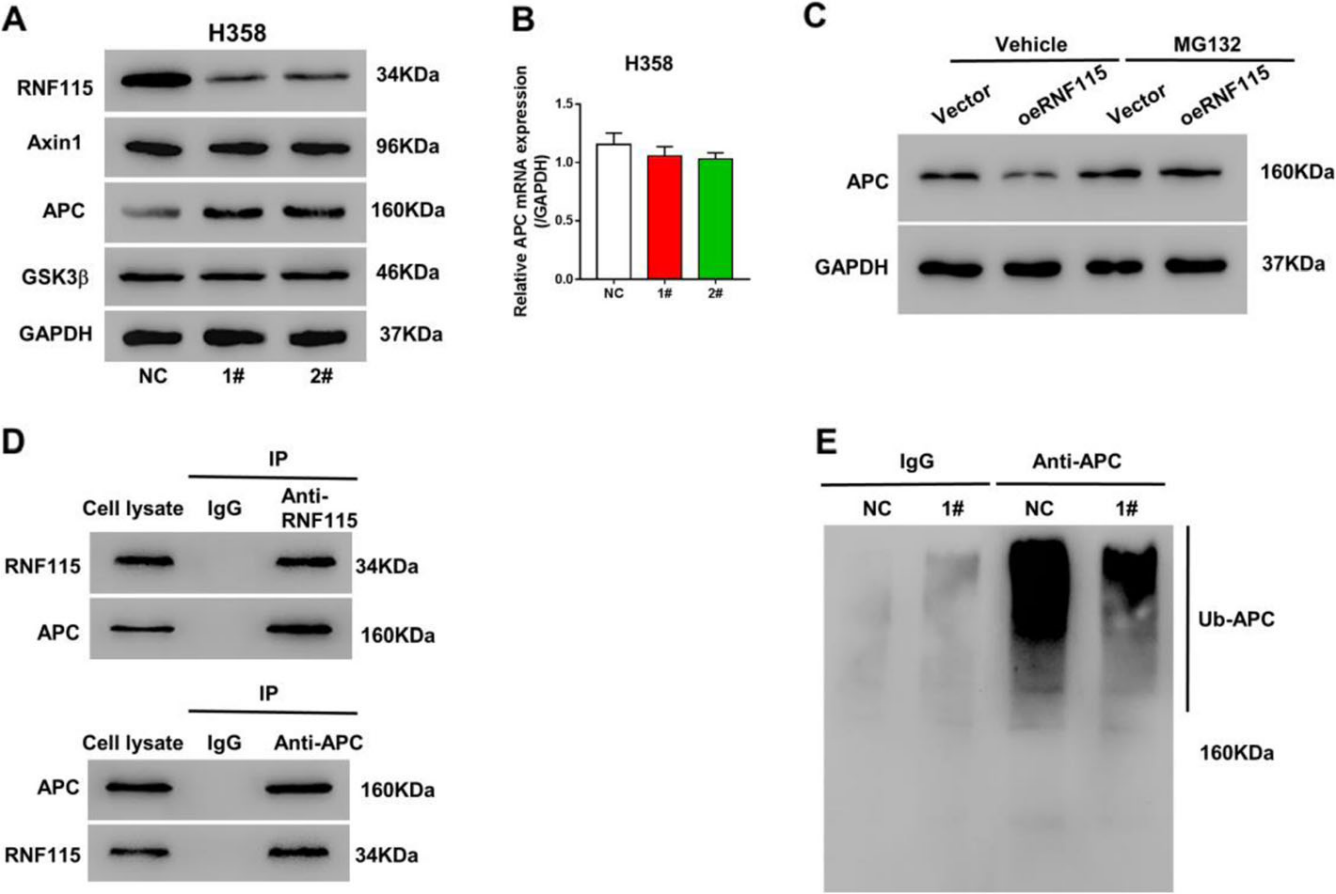
Effects of RNF115 on β-catenin regulators and APC ubiquitination in LUAD cells. **a** Expressions of RNF115, Axin1, APC, and GSK3β proteins in H358 cells stably expressing shRNF115 (1# and 2#) or control shRNA (NC) by western blotting with the loading control as GAPDH. **b** The transcriptional levels of APC in H358 cells stably expressing shRNF115 (1# and 2#) or control shRNA (NC) by qRT-PCR. **c** H1299 cells transfected with oeRNF115 or Vector control were treated by MG132 or DMSO (vehicle). APC expression was detected by western blotting. **d** Association between RNF115 and APC by co-immunoprecipitation. **e** Immunoprecipitation and immunoblotting of APC in H358 cells stably expressing shRNF115 (1#) or control shRNA (NC)

To investigate the potential mechanism by which RNF115 regulates β-catenin pathway in LUAD cells, we further analyzed the levels of multiple β-catenin regulator protein expressions, including Axin1, APC, and GSK3β. We observed that the APC expression was elevated in H358 cells with RNF115 knockdown (both shRNF115 1# and 2#) in comparison with the control, while the expression levels of Axin1 and GSK3β were not noticeably changed (Fig. 5a). However, no significant (*P* > 0.05) alteration in APC was observed in H358 cells with RNF115 knockdown (either shRNF115 1# or 2#) at the transcriptional level, when it was compared to the control cells (Fig. 5b). Therefore, we speculated that the effect of RNF115 on the expression of APC mainly depended on regulation at the protein level. RNF115 overexpression reduced the protein level of APC in H1299 cells, which was suppressed a proteasome inhibitor, MG132 (Fig. 5c). As a matter of fact, through Co-IP assay, we actually detected the association between RNF115 and APC proteins (Fig. 5d). Moreover, based on the fact that RNF115 is an ubiquitin ligase, we also speculated that RNF115 could regulate APC through ubiquitination. As a result, we evaluated its activity in APC ubiquitination by immunoprecipitation and immunoblotting, through which we found that RNF115 knockdown in H358 cells (shRNF115 1#) inhibited the cellular APC ubiquitination (Fig. 5e).

**Fig. 6:**
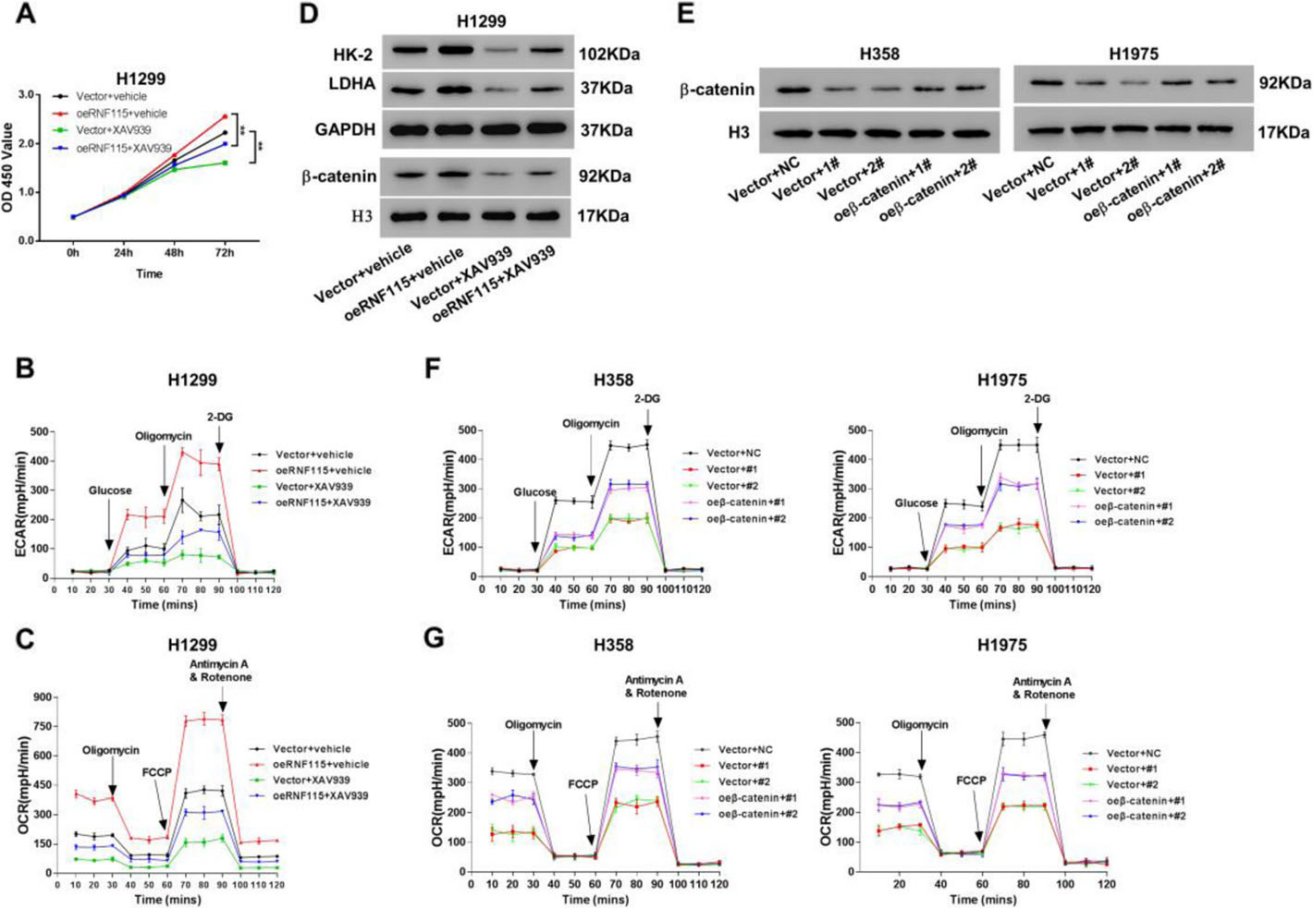
Involvement of β-catenin pathway in the biological functions of RNF115 in LUAD cells. **a**–**d** H1299 cells transfected with oeRNF115 or Vector control were treated by XAV939 or DMSO (vehicle). **a** Measurement of cell proliferation based on CCK-8. **b** Extracellular acidification rates for glycolysis. **c** Oxygen consumption rates for mitochondrial respiration. **d** The protein expressions of cytoplasmic HK-2 and LDHA with GAPDH as the loading control and β-catenin in nucleus with H3 as the loading control by western blotting. **e**–**g** H358 or H1975 cells transfected with shRNF115 (1# and 2#) or control shRNA (NC) and oeβ-catemom or Vector control. **e** Expression of β-catenin in nucleus by western blotting with the loading control as H3. **f** Extracellular acidification rates for glycolysis. **g** Oxygen consumption rates for mitochondrial respiration **P < 0.01

To further validate that RNF115 regulates cellular activities in LUAD through modulating Wnt/β-catenin pathway, we used β-catenin inhibitor XAV939 as a treatment for H1299 cells with RNF115 overexpression (oeRNF115) (Fig. 6a–e), and overexpressed β-catenin in H358 and H1975 cells with RNF115 knockdown (Fig. 6f–h). Overall, we have observed that the cellular proliferation (Fig. 6a), glycolytic activities (Fig. 6b), mitochondrial respiration (Fig. 6c), and related protein (HK-2 and LDHA) expression levels (Fig. 6d) of H1299 cells were all diminished by XAV939, compared with the respective control. Furthermore, the β-catenin inhibitor XAV939 counteracted the stimulatory effects of RNF115 overexpression on all these features mentioned above. On the contrary, overexpression of β-catenin counteracted the RNF115 knockdown on glycolytic activities (Fig. 6g) and mitochondrial respiration (Fig. 6h). These data suggested that RNF115 modulated metabolic processes through regulating β-catenin pathway.

## Discussion

Proteins containing RING finger domain are the major and crucial facilitator of ubiquitin-mediated proteasomal degradation, by which they contribute to modulate various biological cellular activities and regulate oncogenesis in cells [20, 28]. For the current study, we focused on RNF115 since its BCA2 zinc-finger domain, once activated, is capable of catalyzing auto-ubiquitination of interactive proteins [29]. Through analyzing the patient data collected from both TCGA database (Fig. 1a) and Shanghai Chest Hospital, Shanghai Jiao Tong University (Fig. 1c–f), we observed the substantially high expression of RNF115 in tumorous tissues from LUAD patients at both protein and transcriptional levels. Meanwhile, based on bioinformatics analysis and our own cohort, we have also demonstrated the negative correlation between RNF115 expression and overall survival probability in LUAD patients after diagnosis and surgery (Fig. 1b, g), which is correspondent with several previous studies that reported similar connections between RING finger ubiquitin ligases and mortality rates of cancer patients [30–34]. Although further univariate survival analysis is warrant to determine the independency of RNF115 on survival rate of LUAD patients, these analyses indicate that RNF115 expression is firmly associated with LUAD prognosis in clinical management and that RNF115 might serve as one of the prognostic biomarkers for LUAD patients.

The high expression of RNF115 is also connected with more than half of the invasive breast cancer cases, for which it dynamically participates in regulating the cell proliferation and tumor progression in breast cancer [21, 26]. For the present study, we detected that the overexpression of RNF115 in LUAD cells stimulated the cellular proliferation and suppressed the cell apoptosis (Fig. 2f–i), while on the contrary, RNF115 knockdown in LUAD cells showed the reverse effects (Fig. 2a–d), which could be rescued by overexpressing of RNAi-resistant mutant of RNF115 (Figure S1A-B). Furthermore, xenografts experiments confirmed the in vitro data (Fig. 3). Additionally, ectopic expression of RNF115 in 16HBE cells to the level of H358 cells resulted in a comparable proliferation rate to H358 cells (Figure S3A-B). These data indicates that RNF115 could promote LUAD activities through promoting cell proliferation and inhibiting apoptotic processes.

Furthermore, RING finger ubiquitin ligases modulate the metabolic activities in both normal and tumorous cells via regulating metabolism-related protein factors [35, 36]. Here we also investigated the effect of RNF115 on LUAD cellular metabolic processes. A positive correlation was revealed between RNF115 expression and glycolytic activity or β-catenin pathway (Fig. 4a). We have found that RNF115 knockdown in LUAD cells reduced their glycolytic process and mitochondrial respiration, which was rescued by overexpressing of RNAi-resistant mutant of RNF115 (Figure S1C-D). Additionally, ectopic expression of RNF115 in 16HBE cells to the level of H358 cells resulted in a comparable glycolytic process and mitochondrial respiration to H358 cells (Figure S3C-D). All of these results indicate the stimulatory effect of RNF115 on the overall metabolic activities of LUAD cells, and this function is possibly through RNF115 in regulating β-catenin pathway. However, this conclusion could be further validated by examining the effect of RNF115 overexpression in LUAD cellular metabolic processes in future study.

Wnt signaling pathway plays essential parts in the regulation of cell cycle and differentiation, and the deregulation of its canonical (β-catenin-dependent) pathway has greater implications for the initiation and progression of various types of human cancers [37]. Previously, RING finger ubiquitin ligases have been reported to affect the stemness and metastasis of gastrointestinal related cancers through regulating Wnt/β-catenin signaling pathway [38–41]. In accordance with these findings, we also revealed that the underlying mechanism of the LUAD-promotive functions of RNF115, found in this study, is at least partially via modulating Wnt canonical signaling pathway. The knockdown of RNF115 in LUAD cells induced no alteration on APC gene expression, but it could upregulate the cytoplasmic protein level of APC and downregulate the nuclear protein level of β-catenin (Figs. 4e and 5a, b), indicating the bio-function of RNF115 in limiting APC at protein level and inducing the nuclear accretion of β-catenin. As a matter of fact, APC is one of the essential components forming the destruction complex at Wnt-off-state, which initiates the phosphorylation and ubiquitin-mediated proteasomal degradation of cytoplasmic β-catenin and blocks the accumulation of β-catenin in the nucleus for preventing the mediation of potential oncogenesis in normal cells [42].

The elevated protein level of APC in the cytosol induced by RNF115 knockdown in LUAD cells was found to be connected with the diminished degree of its ubiquitination (Fig. 5c, d). This finding further demonstrates that the functional role of RNF115 in regulating Wnt/β-catenin pathway is through catalyzing the ubiquitination of β-catenin regulator APC, which is supported by its biochemical characteristic as E3 ubiquitin ligase [29, 43]. Additionally, the simultaneous treatment of β-catenin inhibitor or overexpression of β-catenin could counteract the promotive effect of RNF115 overexpression in LUAD cellular activities (Fig. 6), which further confirms the participation of RNF115 in tumor cell proliferation, programed cell death, and energy metabolism via Wnt/β-catenin signaling pathway. Thus, we have revealed the comprehensive biological function of RNF115 in promoting LUAD activities by regulating Wnt canonical pathway through blocking β-catenin accumulation in the cells via mediating the ubiquitination of cytoplasmic APC.

## Conclusion

To our knowledge, this study is original in revealing the bio-function of RNF115 in promoting lung cancer. Conclusively, we demonstrate the high expression of RNF115 in LUAD, its significant correlation with LUAD prognosis, and the stimulatory effects of RNF115 on LUAD activities in terms of their cell proliferation, programed cell death, and cellular metabolic processes. Moreover, we also suggest that the underlying mechanism of the biological function of RNF115 in LUAD is potentially through the regulation of Wnt/β-catenin signaling pathway by mediating APC ubiquitination. Considering all of these findings, this study is prominent in paving the avenue for further development of therapeutic targets and prognostic markers for lung cancer, especially LUAD.

## Supplementary Information

**Additional file 1: Table S1. Antibody list.** **Figure S1.** Expressions of RNF115 protein in various cell lines. (A) RNF115 protein expressions in 16HBE cells and five LUAD cell lines. GAPDH as loading control. (B) RNF115 protein expressions in H358 or H1975 cells transfected with shRNF115 (1#, 2#, and 3#), shRNA (NC), and non-transfected cells (control) by western blotting. The loading control as GAPDH. (C) RNF115 protein expressions in H1299 cells transfected with oeRNF115, Vector, and non-transfected cells (control). The loading control as GAPDH. **Figure S2.** RNAi resistant mutant of RNF115 rescued the effects of RNF115 shRNA on cell proliferation and cellular metabolism in LUAD cells. H358 and H1975 cells were transfected with shRNF115 (1# and 2#) or control shRNA (shNC), and transfected with plasmids expressing RNAi resistant mutant RNF115 (RrRNA115). (A) RNF115 expression was detected by western blotting. (B) Measurement of cell proliferation based on CCK-8. (C) Extracellular acidification rates for glycolysis. (D) Oxygen consumption rates for mitochondrial respiration. **P<0.01. **Figure S3.** Overexpression of RNF115 promoted cell proliferation, glycolysis and mitochondrial respiration in 16HBE cells. (A) RNF115 expression was detected by western blotting. (B) Measurement of cell proliferation based on CCK-8. (C) The ratio of apoptotic cells. (D) Extracellular acidification rates for glycolysis. (E) Oxygen consumption rates for mitochondrial respiration.

## Data Availability

The data of this study are available from the corresponding authors for reasonable requests.

## Abbreviations

NSCLC: Non-small cell lung cancer
LUAD: Lung adenocarcinoma
APC: Adenomatous polyposis coli
AXIN: Axis inhibition protein
GSK-3β: Glycogen synthase kinase 3β
RNF: RING finger protein
GSEA: Gene Set Enrichment Analysis
TCGA: The Cancer Genome Atlas
qRT-PCR: Quantitative real-time PCR
IHC: Immunohistochemistry
