# Supplementary material for: RNF115 promotes lung adenocarcinoma through Wnt/β-catenin pathway activation by mediating APC ubiquitination
Source: Cancer Metab. 2021 Jan 28;9:7. doi: 10.1186/s40170-021-00243-y (PMC7842072; doi:10.1186/s40170-021-00243-y)
Supplement: Supplementary file 1 — Additional file 1: Table S1. Antibody list. Figure S1. Expressions of RNF115 protein in various cell lines. (A) RNF115 protein expressions in 16HBE cells and five LUAD cell lines. GAPDH as loading control. (B) RNF115 protein expressions in H358 or H1975 cells transfected with shRNF115 (1#, 2#, and 3#), shRNA (NC), and non-transfected cells (control) by western blotting. The loading control as GAPDH. (C) RNF115 protein expressions in H1299 cells transfected with oeRNF115, Vector, and non-transfected cells (control). The loading control as GAPDH. Figure S2. RNAi resistant mutant of RNF115 rescued the effects of RNF115 shRNA on cell proliferation and cellular metabolism in LUAD cells. H358 and H1975 cells were transfected with shRNF115 (1# and 2#) or control shRNA (shNC), and transfected with plasmids expressing RNAi resistant mutant RNF115 (RrRNA115). (A) RNF115 expression was detected by western blotting. (B) Measurement of cell proliferation based on CCK-8. (C) Extracellular acidification rates for glycolysis. (D) Oxygen consumption rates for mitochondrial respiration. **P<0.01. Figure S3. Overexpression of RNF115 promoted cell proliferation, glycolysis and mitochondrial respiration in 16HBE cells. (A) RNF115 expression was detected by western blotting. (B) Measurement of cell proliferation based on CCK-8. (C) The ratio of apoptotic cells. (D) Extracellular acidification rates for glycolysis. (E) Oxygen consumption rates for mitochondrial respiration. [file 40170_2021_243_MOESM1_ESM.docx]

**Table S1.** Antibody list.

| **Primary antibody** | **Company** | **Catalog No.** |
| --- | --- | --- |
| RNF115 | Abcam | Ab187642 |
| PCNA | Abcam | Ab92552 |
| Cleaved caspase-3 | Abcam | Ab2302 |
| HK2 | Cell Signaling Technology | #2867 |
| LDHA | Abcam | Ab125683 |
| β-catenin | Cell Signaling Technology | #9587 |
| APC | Cell Signaling Technology | #2504 |
| Axin1 | Affinity | DF9264 |
| GSK-3β | Cell Signaling Technology | #12456 |
| GAPDH | Cell Signaling Technology | #5174 |
| Ubiquitin | Abcam | Ab7780 |

**
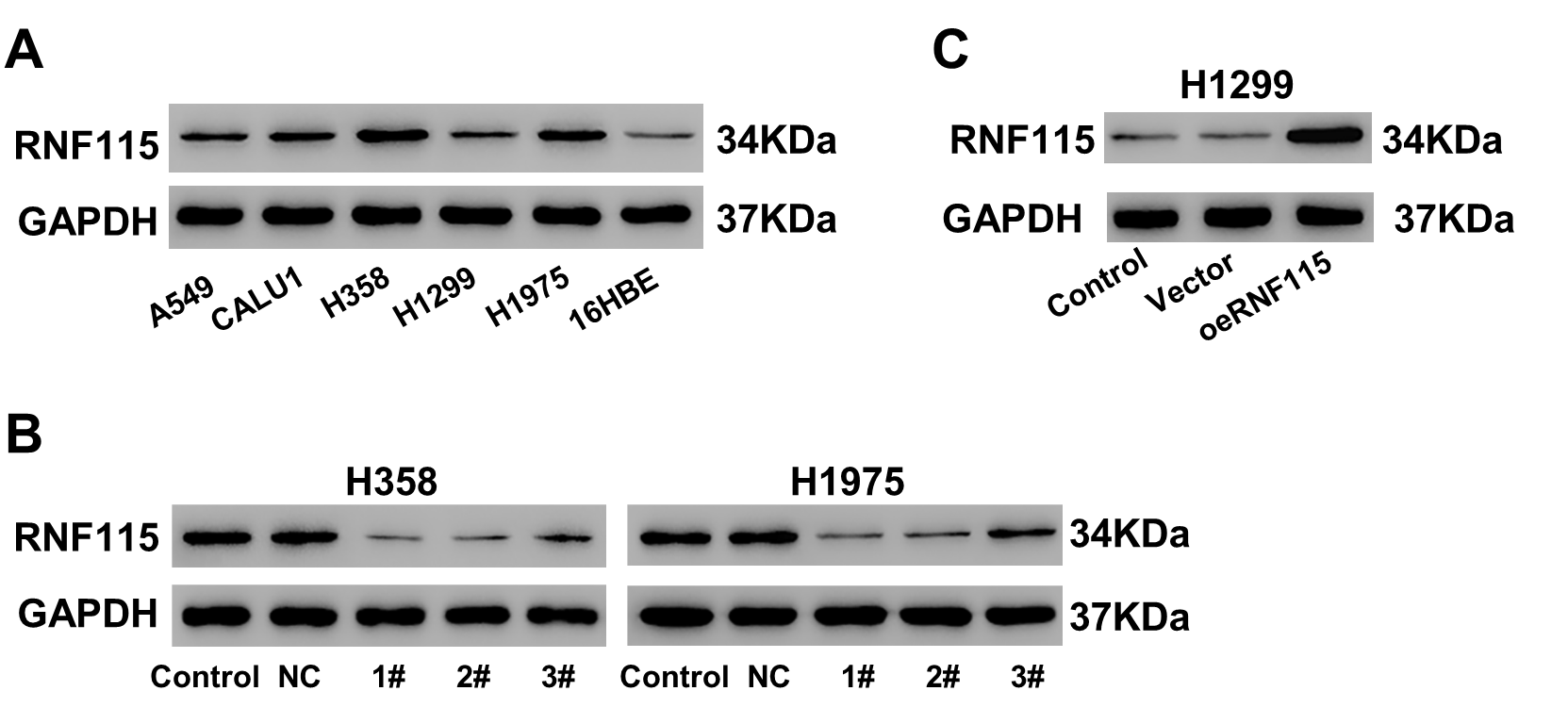
**

**Figure S1.** Expressions of RNF115 protein in various cell lines. (A) RNF115 protein expressions in 16HBE cells and five LUAD cell lines. GAPDH as loading control. (B) RNF115 protein expressions in H358 or H1975 cells transfected with shRNF115 (1#, 2#, and 3#), shRNA (NC), and non-transfected cells (control) by western blotting. The loading control as GAPDH. (C) RNF115 protein expressions in H1299 cells transfected with oeRNF115, Vector, and non-transfected cells (control). The loading control as GAPDH.

**
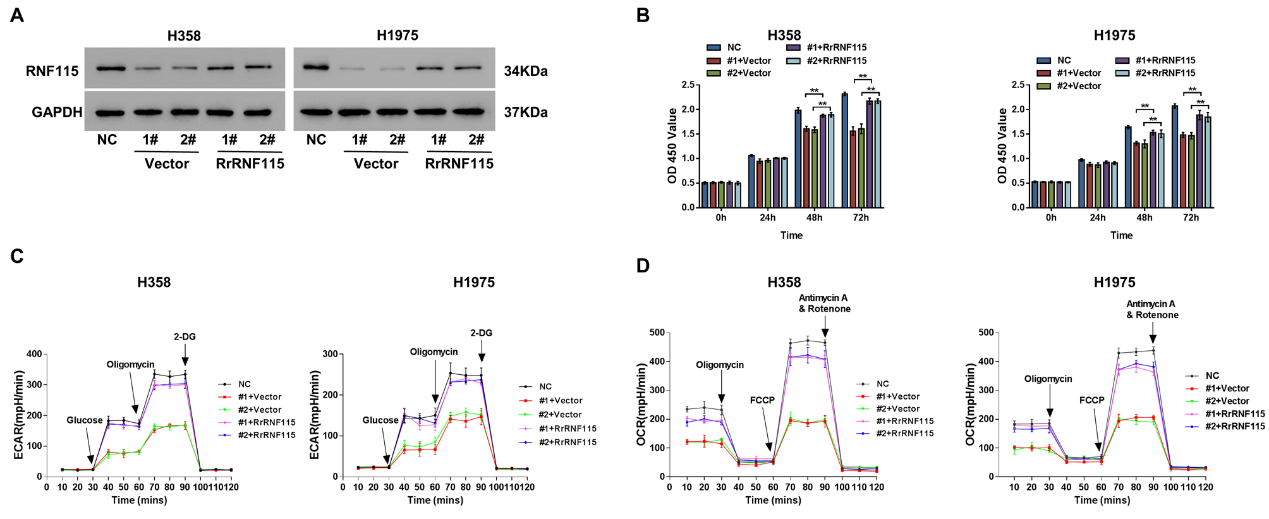
**

**Figure S2.** RNAi resistant mutant of RNF115 rescued the effects of RNF115 shRNA on cell proliferation and cellular metabolism in LUAD cells. H358 and H1975 cells were transfected with shRNF115 (1# and 2#) or control shRNA (shNC), and transfected with plasmids expressing RNAi resistant mutant RNF115 (RrRNA115). (A) RNF115 expression was detected by western blotting. (B) Measurement of cell proliferation based on CCK-8. (C) Extracellular acidification rates for glycolysis. (D) Oxygen consumption rates for mitochondrial respiration. **P<0.01.


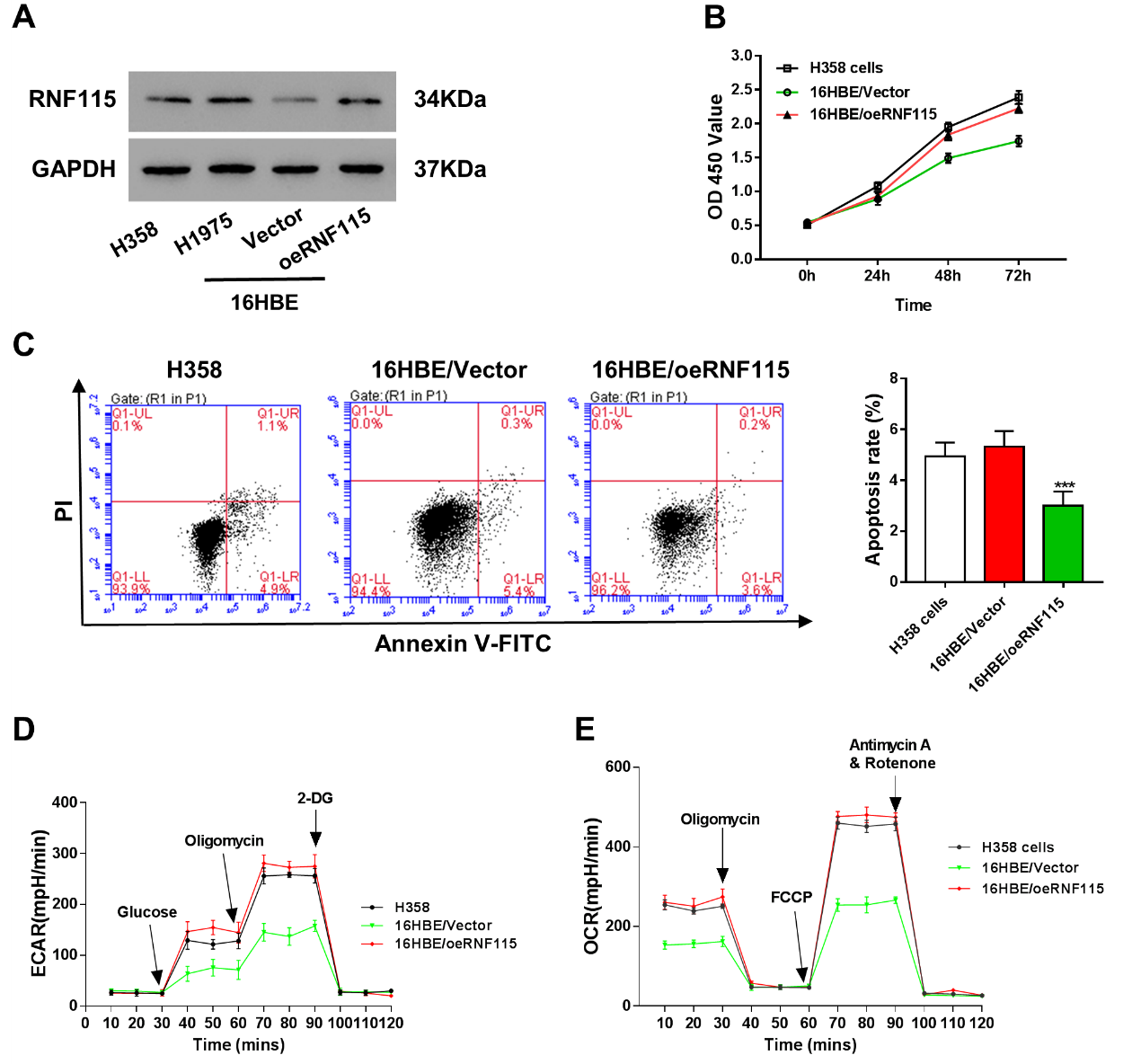


**Figure S3.** Overexpression of RNF115 promoted cell proliferation, glycolysis and mitochondrial respiration in 16HBE cells. (A) RNF115 expression was detected by western blotting. (B) Measurement of cell proliferation based on CCK-8. (C) The ratio of apoptotic cells. (D) Extracellular acidification rates for glycolysis. (E) Oxygen consumption rates for mitochondrial respiration.
